# Gamma gap thresholds and HIV, hepatitis C, and monoclonal gammopathy

**DOI:** 10.1371/journal.pone.0224977

**Published:** 2020-01-15

**Authors:** Gigi Y. Liu, Olive Tang, Daniel J. Brotman, Edgar R. Miller, Alison R. Moliterno, Stephen P. Juraschek

**Affiliations:** 1 Division of General Internal Medicine, Johns Hopkins University School of Medicine, Baltimore, Maryland, United States of America; 2 The Johns Hopkins Bloomberg School of Public Health Department of Epidemiology, and The Welch Center for Prevention, Epidemiology and Clinical Research, Baltimore, Maryland, United States of America; 3 Division of Hematology, Johns Hopkins University School of Medicine, Baltimore, Maryland, United States of America; 4 Beth Israel Deaconess Medical Center, Harvard Medical School, Boston, Massachusetts, United States of America; Inserm U0152, UMR 5286, FRANCE

## Abstract

**Background:**

An elevated gamma gap (>4 g/dL), the difference between serum total protein and albumin, can trigger testing for chronic infections or monoclonal gammopathy, despite a lack of evidence supporting this clinical threshold.

**Methods:**

Using the National Health and Nutrition Examination Survey (NHANES) 1999–2014, gamma gap was derived in three subpopulations based on availability of testing for human immunodeficiency virus (HIV; N = 25,680), hepatitis C (HCV; N = 45,134), and monoclonal gammopathy of unknown significance (MGUS; N = 6,118). Disease status was confirmed by HIV antibody and Western blot, HCV RNA test, or electrophoresis with immunofixation. Sensitivity, specificity, and likelihood ratios were calculated for different gamma gap thresholds. Area under the curve (AUC) was used to assess performance and cubic splines were used to characterize the relationship between the gamma gap and each disease.

**Results:**

Mean gamma gaps of participants with HIV, HCV, or MGUS ranged from 3.4–3.8 g/dL. The AUC was 0.80 (95%CI: 0.75,0.85) for HIV, 0.74 (0.72,0.76) for HCV, and 0.64 (0.60,0.69) for MGUS. An elevated gamma gap of over 4 g/dL corresponded to sensitivities of 39.3%, 19.0%, and 15.4% and specificities of 98.4%, 97.8%, and 95.4% for HIV, HCV, and MGUS, respectively. A higher prevalence of all three diseases was observed at both low and high gamma gaps.

**Discussion:**

An elevated gamma gap of 4 g/dL is insensitive for HIV, HCV, or MGUS, but has a high specificity for HIV and HCV, suggesting that the absence of an elevated gamma gap does not rule out HIV, HCV, or MGUS. Conversely, an elevated gap may justify further testing for HIV and HCV, but does not justify electrophoresis in the absence of additional clinical information.

## Introduction

The gamma gap, sometimes referred to as a protein gap, is the difference between total serum protein and serum albumin, which is readily available through a frequently obtained comprehensive metabolic panel (CMP) [1]. It represents serum proteins other than albumin, including α1, α2, β, and γ globulins [2]. This gap is associated with a variety of inflammatory and infectious conditions [3–7] and serum acute phase reactants, such as haptoglobin and C-reactive protein [2,8]. Furthermore, it is an independent risk factor for all-cause mortality [9,10] even after adjustment for cardiovascular, pulmonary, and oncologic diseases [4]. Clinically, an arbitrary value of 4.0 g/dL is sometimes considered an elevated gamma gap even though there are no studies demonstrating an association between an elevated gamma gap and various clinical conditions [4,11]. Nevertheless, an elevated gamma gap of 4.0 g/dL may trigger further work-up such as serum electrophoresis and free light chain assessment despite a lack of published evidence informing this testing [11–15]. Similarly, Western blots for HIV or serologic antigen and antibody testing for HCV may be performed in response to an elevated gamma gap.

Despite the clinical significance of the gamma gap and its widespread use, there are no agreed upon thresholds to inform the decision of conducting further testing. In fact, the few studies that exist suggest a low yield from elevated gamma gap as the sole rationale for further workup. Two studies showed that among patients with a gamma gap >4 g/dl, less than 1% had monoclonal gammopathy or paraproteinemia ascribed to MGUS or lymphoproliferative disorders [16,17]. Furthermore, the utility of the gamma gap as a screening test for associated conditions has not been established, which may contribute to inefficient, unnecessary and potentially costly testing.

Here, we examine the performance of the gamma gap measured in a community-based population as a diagnostic test for conditions thought to be associated with an elevated gamma gap, namely, human immunodeficiency virus (HIV), hepatitis C (HCV), and monoclonal gammopathy of unknown significance (MGUS).

## Materials and methods

### Study population

The Continuous National Health and Nutrition Examination Surveys (NHANES) are a series of cross-sectional, stratified, clustered probability national health surveys conducted by the National Center for Health Statistics (NCHS) in two-year cycles, with samples that are representative of the non-institutionalized U.S. population. The NHANES include ancillary tests to their surveys that vary from cycle to cycle, based on sponsorship or current objectives of the Centers for Disease Control and Prevention. As a result, the study population for each of the following conditions varied based on test administration within certain cycles. For the purposes of our analyses, the subset of participants tested for each of the following conditions–HIV, HCV, and MGUS–were treated as distinct populations nested within the broader NHANES sample. Protocols for the administration and conduct of these studies were approved by the NCHS institutional review board and informed consent was obtained from all participants. All data were fully de-identified prior to being made publicly available.

### Gamma gap

The gamma gap was determined using the laboratory data available in the Standard Biochemistry Profile, which was conducted for all participants in NHANES 1999–2014. Between 1999 and 2006, serum albumin was measured using the Boehringer Mannheim Diagnostic system (now Roche Diagnostic Corporation, Indianapolis, Indiana) and then from 2007 onward using the bichromatic Dcx800 assay (Beckman Coulter, Inc., Indianapolis, Indiana). Total protein was measured in serum using a colorimetric assay through 2002 and then was switched to the Beckman Synchron LX20 through 2008. Measurements of total protein were done using the Dcx800 system from 2009 onward [18]. Albumin was subtracted from total protein to calculate the gamma gap. This was then rounded to the nearest tenth digit for analysis.

### HIV testing

All 18–59 year-old participants between 1999–2014 were eligible, with 25,680 ultimately undergoing HIV testing (N = 25,680). This population was selected by NHANES based on known patterns of the distribution of HIV infection in the general population. For all of these surveys, HIV testing was conducted using the Synthetic Peptide Enzyme Immunoassay (EIA) from Genetic Systems HIV-1/HIB-2 Peptide EIA and confirmed by Western blot [19]. A positive HIV status required both tests to be positive.

### HCV testing

All participants over the age of 6 between 1999–2014 were eligible, with 45,134 ultimately undergoing HCV testing. Participants were screened for antibodies to HCV core antigen. Then, anyone with a positive HCV core antibody underwent a HCV-RNA test, which was detected using the COBAS AMPLICOR HCV MONITOR Test version 2.0 [20,21]. A positive HCV status was defined as a positive HCV-RNA test. Exposure to HCV was based on the presence of antibodies measured in serum specimens, using the Ortho HCV enzyme-linked immunosorbent assay (ELISA), version 3.0 (Ortho-Clinical Diagnostics, Raritan, New Jersey). All ELISA-positive specimens were confirmed via a recombinant immunoblot assay (RIBA HCV 3.0 Strip Immunoblot Assay, Chiron, Emeryville, California). Participants were required to be positive on both tests to be considered as having been infected with HCV. Participants with negative RIBA results, were considered anti-HCV negative. Positive and indeterminate cases were assessed for HCV RNA (viral load) using an in vitro nucleic acid amplification test for the quantitation of HCV RNA: COBAS AMPLICOR HCV Test (survey 2005–14), and COBAS AmpliPrep/ TaqMan HCV Test, version 2.0 (survey years 1999–2004). Participants were considered as having a chronic HCV infection if both the anti-HCV ELISA and RIBA tests were confirmed positive or indeterminate and if the results of their test for HCV RNA test were positive.

### Monoclonal gammopathy of unknown significance

Electrophoresis to assess for monoclonal gammopathy of unknown significance (MGUS) was performed in participants over the age of 50 between 1999–2004 (N = 6,118). Serum samples were first analyzed via agarose gel electrophoresis and then retested with immunofixation for validation. MGUS was considered positive based on the presence of either heavy or light chains Testing was conducted at the Protein Immunology Laboratory at Mayo Clinic in Rochester, MN [22].

### Other covariates

Demographic data were self-reported by study participants: age, sex, race/ethnicity (categorized as recommended by the CDC/NCHS as Non-Hispanic White, Non-Hispanic Black, Mexican-American, Hispanic, or Other). The following biomarkers were measured as part of a comprehensive metabolic panel: total calcium, aspartate aminotransferase, alanine aminotransferase, alkaline phosphatase, and total bilirubin.

### Statistical analysis

Means and proportions (with standard deviations) were used to characterize participants by disease status for each of the corresponding survey populations. Area under the curve (AUC) was calculated from receiver operating curves (ROC) based on logistic regression to determine the overall discriminatory performance of the gamma gap for each outcome of interest. AUCs were determined for each item of the comprehensive metabolic panel. We also determined sensitivity, specificity, positive likelihood ratio, and negative likelihood ratio for each of the different gamma gap thresholds for HIV, HCV, and MGUS. Poisson regression with cubic splines was used to model prevalence ratios, characterizing the relationship between the gamma gap and each condition. Both logistic and Poisson regressions were adjusted for age, sex, and race. All statistical analyses were conducted using Stata 14.0 (StataCorp).

## Results

Our study included subpopulations of NHANES for HIV (N = 25,680 during 1999 to 2014), HCV (N = 45,134 during 1999 to 2014), and MGUS (N = 6,118 during 1999 to 2004). Demographic characteristics are shown in **Table 1**. Across all subpopulations, approximately 50% of participants were women. Compared to the HIV and HCV subpopulations, the MGUS group had a higher proportion of white participants (57.7%) and a higher mean age of 67 years (SD = 10.5). The mean gamma gap of participants positive for the three diseases ranged from 3.4 g/dL to 3.8 g/dL, which were higher than those without these diseases (3.0 g/dL to 3.1 g/dL). Of those positive for HIV, 37.8% of them had an elevated gamma gap of 4 g/dL, compared to 18.8% for HCV and 15.4% for MGUS. The median (25^th^ and 75^th^ quartiles of gamma gap for each of the three conditions was 2.9 (2.7, 3.2) g/dL, 3.0 (2.7, 3.7) g/dL, and 3.1 (2.8, 3.4) g/dL, for HIV, HCV, and MGUS, respectively.

**Table 1:** Baseline characteristics.

	HIV			HCV			MGUS		
	Overall	Negative	Positive	Overall	Negative	Positive	Overall	Negative	Positive
	*(n = 25,680)*	*(n = 25,545)*	*(n = 135)*	*(n = 45,134)*	*(n = 44,676)*	*(n = 458)*	*(n = 6,118)*	*(n = 5,949)*	*(n = 169)*
**Age, yr (SD)**	34.3 (11.3)	34.3 (11.4)	38.9 (9.6)	40.7 (21.8)	40.6 (21.8)	51.7 (11.6)	66.9 (10.5)	66.8 (10.5)	72.0 (9.5)
**Women, %**	13459 (52.4)	13425 (52.6)	34 (25.2)	22997 (51.0)	22849 (51.1)	148 (32.3)	3090 (50.5)	3021 (50.8)	69 (40.8)
**Race, %**									
** Non-Hispanic White**	10381 (40.4)	10361 (40.6)	20 (14.8)	19145 (42.4)	18974 (42.5)	171 (37.3)	3529 (57.7)	3429 (57.6)	100 (59.2)
** Non-Hispanic Black**	5576 (21.7)	5484 (21.5)	92 (68.1)	10130 (22.4)	9944 (22.3)	186 (40.6)	977 (16.0)	937 (15.8)	40 (23.7)
** Mexican American**	5539 (21.6)	5525 (21.6)	14 (10.4)	10051 (22.3)	9998 (22.4)	53 (11.6)	1194 (19.5)	1172 (19.7)	22 (13.0)
** Hispanic Other**	2029 (7.9)	2021 (7.9)	8 (5.9)	3153 (7.0)	3121 (7.0)	32 (7.0)	237 (3.9)	235 (4.0)	2 (1.2)
** Non-Hispanic Other**	2155 (8.4)	2154 (8.4)	1 (0.7)	2655 (5.9)	2639 (5.9)	16 (3.5)	181 (3.0)	176 (3.0)	5 (3.0)
**Albumin, g/dL (SD)**	4.3 (0.4)	4.3 (0.4)	4.1 (0.4)	4.3 (0.4)	4.3 (0.4)	4.1 (0.4)	4.2 (0.3)	4.2 (0.3)	4.1 (0.3)
**Total Protein, g/dL**	7.2 (0.5)	7.2 (0.5)	7.9 (0.8)	7.3 (0.5)	7.3 (0.5)	7.5 (0.6)	7.3 (0.5)	7.3 (0.5)	7.5 (0.7)
**Gamma Gap, g/dL (SD)**	3.0 (0.4)	3.0 (0.4)	3.8 (0.9)	3.0 (0.4)	3.0 (0.4)	3.5 (0.6)	3.1 (0.5)	3.1 (0.5)	3.4 (0.7)
**Gamma Gap Fraction**	0.4 (0.0)	0.4 (0.0)	0.5 (0.1)	0.4 (0.0)	0.4 (0.0)	0.5 (0.1)	0.4 (0.0)	0.4 (0.0)	0.5 (0.1)
**Gamma Gap ≥ 4 g/dL, %**	446 (1.7)	395 (1.5)	51 (37.8)	1037 (2.3)	951 (2.1)	86 (18.8)	285 (4.7)	259 (4.4)	26 (15.4)
**Gamma Gap Distribution, g/dL**									
** 1st percentile**	2.1	2.1	2.2	2.1	2.1	2.4	2.1	2.1	2.4
** 5th percentile**	2.3	2.3	2.4	2.3	2.3	2.6	2.4	2.4	2.5
** 25th percentile**	2.7	2.7	3.2	2.7	2.7	3.0	2.8	2.8	3.0
** 50th percentile**	2.9	2.9	3.6	3.0	2.9	3.4	3.1	3.1	3.3
** 75th percentile**	3.2	3.2	4.3	3.2	3.2	3.8	3.4	3.4	3.8
** 95th percentile**	3.7	3.7	5.4	3.7	3.7	4.6	3.9	3.9	5.0
** 99th percentile**	4.2	4.1	6.6	4.2	4.2	5.5	4.5	4.5	5.9

HIV testing was conducted in participants between the ages of 18 and 59 from 1999–2014

HCV testing was conducted in participants over the age of 6 from 1999–2014

MGUS testing was conducted in participants over the age of 50 from 1999–2004

**Fig 1:**
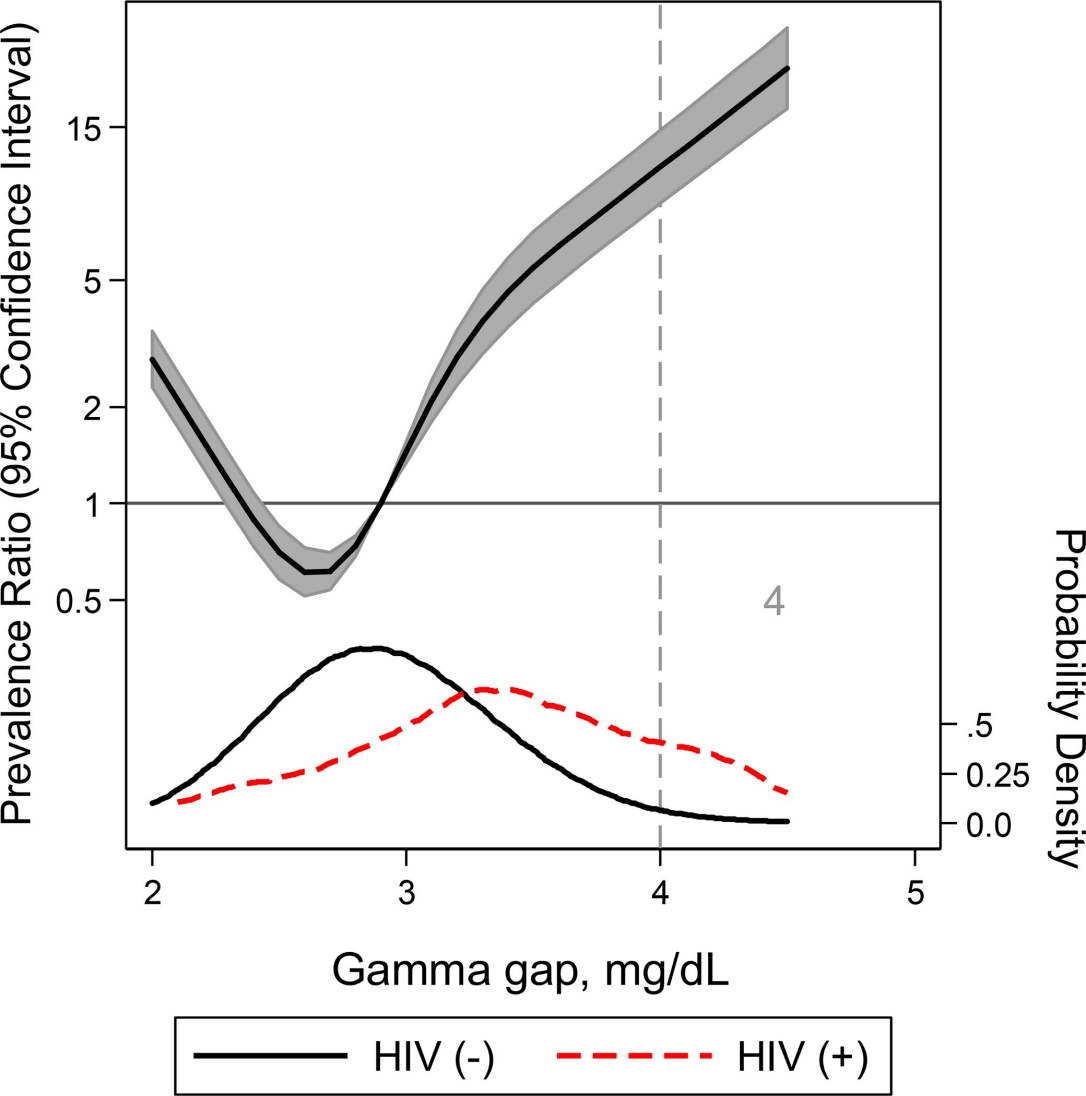
A restricted cubic spline model of the relationship between gamma gap and HIV adjusted for age, gender, and race with three knots. Adjusted prevalence ratio (solid line) and 95% CI were plotted against the gamma gap. Plot was truncated at the 0.5th and 99.5th percentiles. Gamma gap distribution in the case (positive for HIV) sample (red) compared to the control (negative for HIV) sample (black) are also included. The vertical grey line at 4g/dL indicates the commonly used gamma gap cutoff.

For HIV, gamma gap demonstrated an AUC of 0.80 (95% CI: 0.75, 0.85) (**Tables 2 and S1 and S1 Fig**). At the elevated gamma gap threshold of 4 g/dL, the sensitivity and specificity for HIV were 39.3% and 98.4% (LR+ = 24.2; LR- = 0.6) respectively. The continuous association between gamma gap and HIV may be found in **Fig 1**. Notably, the relationship was U-shaped with higher prevalence ratios below 2.5 g/dL as well as above 3.0 g/dL (**Fig 1**).

**Table 2:** Diagnostic performance of gamma gap for HIV using different gamma gap thresholds, N = 25,680.

Gamma gap (g/dL)	Sn, %	Sp, %	LR +	LR -	Overall AUC (95% CI)
≥ 2.5	94.8	10.5	1.1	0.5	0.80 (95% CI: 0.75, 0.85)
≥ 3.0	84.4	51.9	1.8	0.3	
≥ 3.5	59.3	88.1	5.0	0.5	
≥ 4.0	39.3	98.4	24.2	0.6	
≥ 4.5	19.3	99.6	54.1	0.8	
≥ 5.0	10.4	99.9	139.4	0.9	
≥ 5.5	4.4	100.0	283.9	1.0	

**Fig 2:**
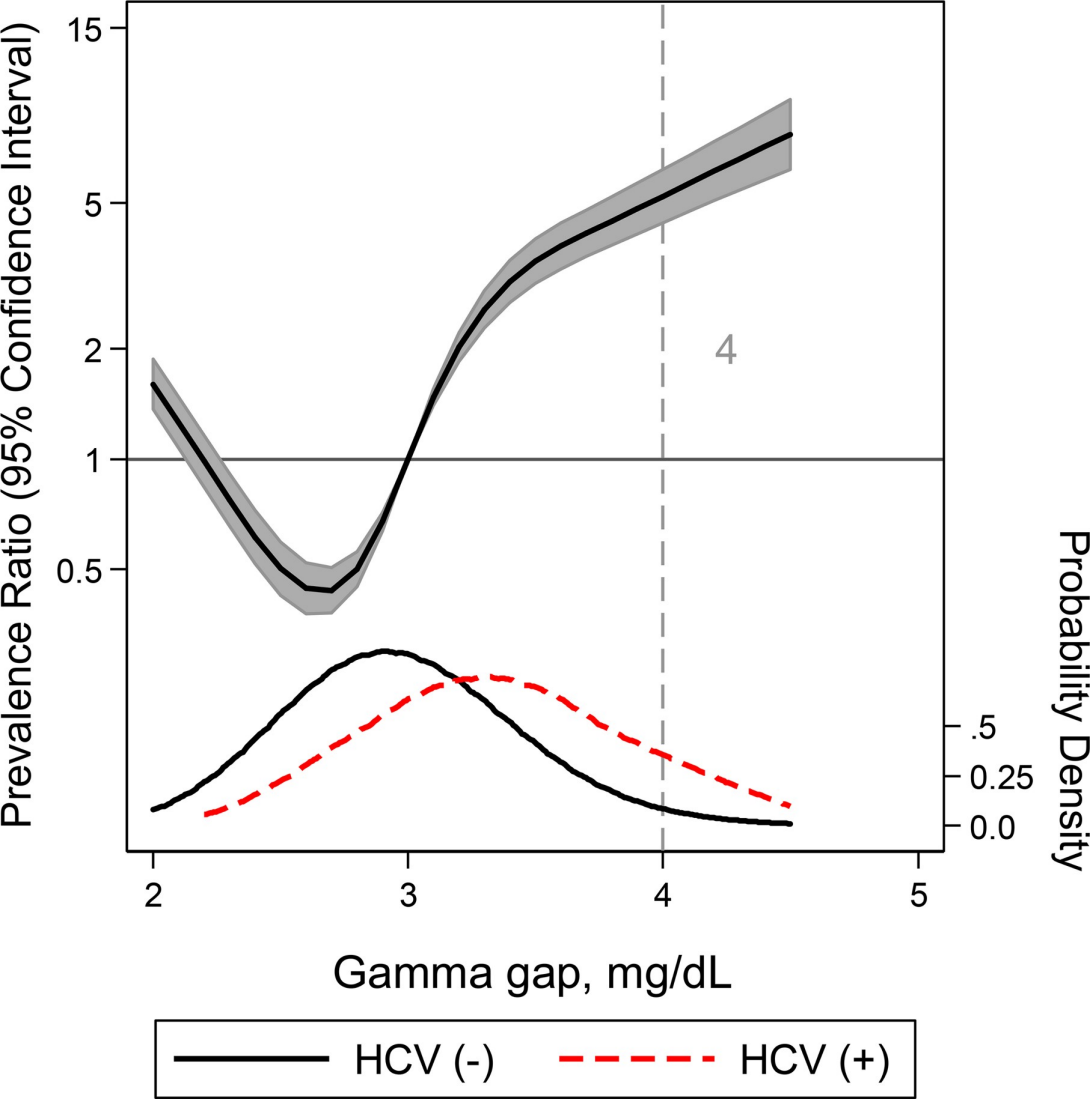
A restricted cubic spline model of the relationship between gamma gap and HCV adjusted for age, gender, and race with three knots. Adjusted prevalence ratio (solid line) and 95% CI were plotted against the gamma gap. Plot was truncated at the 0.5th and 99.5th percentiles. Gamma gap distribution in the case (positive for HCV) sample (red) compared to the control (negative for HCV) sample (black) are also included. The vertical grey line at 4g/dL indicates the commonly used gamma gap cutoff.

For HCV, the gamma gap demonstrated an AUC of 0.74 (95% CI: 0.72, 0.76) (**Tables 3 and S2 and S2 Fig**). While an elevated gamma gap of 4.0 g/dL represented a specificity of 97.8%, a gamma gap of 2.5 or lower corresponded with a sensitivity of 97.8% (**Table 3**). Similar to HIV, we observed a non-linear, U-shaped relationship between gamma gap and the risk of having HCV (**Fig 2**).

**Table 3:** Diagnostic performance of gamma gap for HCV using different gamma gap threshold, N = 45,134.

Gamma gap (g/dL)	Sn, %	Sp, %	LR +	LR -	Overall AUC (95% CI)
≥ 2.5	97.8	9.9	1.1	0.2	0.74 (95% CI: 0.72, 0.76)
≥ 3.0	80.8	50.2	1.6	0.4	
≥ 3.5	44.5	86.9	3.4	0.6	
≥ 4.0	19.0	97.8	8.5	0.8	
≥ 4.5	7.0	99.5	13.7	0.9	
≥ 5.0	2.6	99.8	16.3	1.0	
≥ 5.5	1.1	99.9	13.9	1.0	

**Fig 3:**
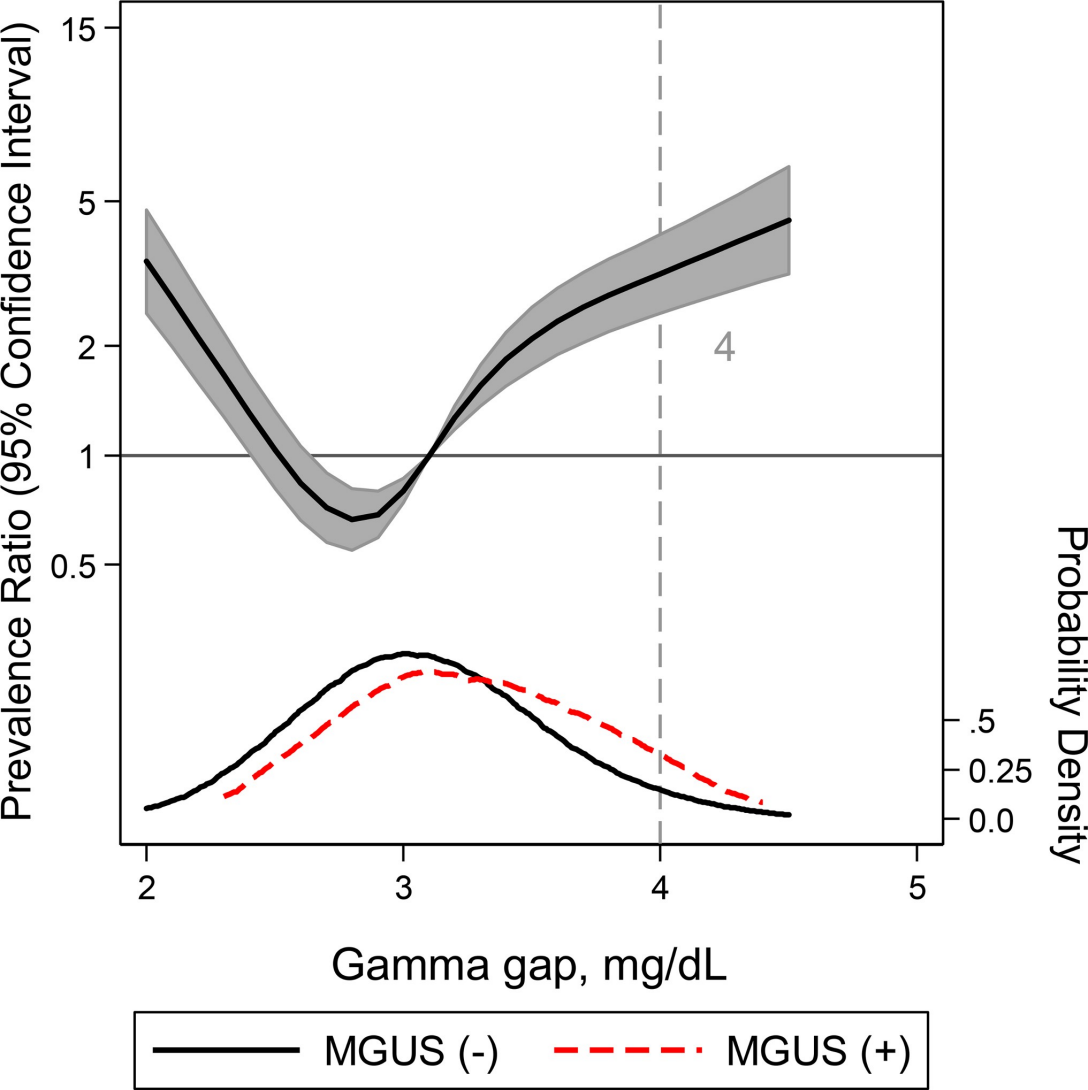
A restricted cubic spline model of the relationship between gamma gap and MGUS adjusted for age, gender, and race with three knots. Adjusted prevalence ratio (solid line) and 95% CI were plotted against the gamma gap. Plot was truncated at the 0.5th and 99.5th percentiles. Gamma gap distribution in the case (positive for MGUS) sample (red) compared to the control (negative for MGUS) sample (black) are also included. The vertical grey line at 4g/dL indicates the commonly used gamma gap cutoff.

For MGUS, gamma gap demonstrated an AUC of 0.64 (95% CI: 0.60, 0.69) (**Tables 4 and S3 and S3 Fig**). At the elevated gamma gap threshold of 4 g/dL, the sensitivity and specificity for MGUS were 15.4% and 95.4% (LR+ = 3.4; LR- = 0.9) respectively. Similar to the preceding conditions, we observed a non-linear, U-shaped relationship between gamma gap and risk of having MGUS (**Fig 3**) as well as a wide distribution of gamma gap resulting in 85% of participants with MGUS having a gamma gap of less than 4 g/dL (**Table 1**).

**Table 4:** Diagnostic performance of gamma gap for MGUS using different gamma gap thresholds, N = 6,118.

Gamma gap (g/dL)	Sn, %	Sp, %	LR +	LR -	Overall AUC (95% CI)
≥ 2.5	97.6	6.4	1.0	0.4	0.64 (95% CI: 0.60, 0.69)
≥ 3.0	75.7	39.9	1.3	0.6	
≥ 3.5	39.1	80.1	2.0	0.8	
≥ 4.0	15.4	95.4	3.4	0.9	
≥ 4.5	7.7	98.9	6.9	0.9	
≥ 5.0	5.3	99.7	16.7	0.9	
≥ 5.5	3.0	99.8	17.6	1.0	

Compared with other items from the comprehensive metabolic panel, the gamma gap demonstrated the highest AUC for HIV and MGUS, but AST and ALT demonstrated the highest AUC for hepatitis C (**S4 Table**).

## Discussion

This study is the first examination of the diagnostic performance of the gamma gap in a general, community-based population for commonly associated conditions. A gamma gap of 4 g/dL demonstrated a high specificity for HIV and HCV, making it useful for increasing the post-test likelihood of having these conditions. However, a threshold of 4 g/dL was insensitive for HIV and HCV, limiting its usefulness in ruling out these conditions. The poor sensitivity of the gamma gap for all three diseases is partially explained by higher prevalence ratios at low gamma gap levels. With regards to MGUS, the gamma gap was neither sensitive nor specific suggesting minimal utility in absence of additional clinical information. As a result, our study does not support use of the gamma gap as the sole rationale for MGUS testing.

In the United States a comprehensive metabolic panel is frequently ordered clinically as a bundle of measurements, which includes total protein, albumin, total calcium, aspartate aminotransferase, alanine aminotransferase, alkaline phosphatase, and total bilirubin. The gamma gap is derived as the difference in total protein and albumin obtained as part of this panel. While the gamma gap is often not the primary purpose of a comprehensive metabolic panel, the incidental finding of a gamma gap may prompt additional diagnostic work-up. There is limited evidence guiding the practice of subsequent testing.

Our study found that an elevated gamma gap demonstrated a high specificity for HIV, which is consistent with HIV’s effect on humoral immunity resulting in hypergammaglobulinemia [23–25]. However, the gamma gap generated by hypergammaglobulinemia is affected by severity of HIV per CD4 count and viral load [26] and its treatment status [25,27,28], which may explain the higher prevalence ratios at lower gamma gap thresholds as well as the poor sensitivity of elevated gamma gap for HIV. Prior studies have shown that regardless of treatment status and viremic level, those with HIV exhibit more intense staining at the gamma region of the serum electrophoresis, represented by antibodies such as IgG and IgA, than those without HIV [27,29], resulting in hypergammaglobulinemia. With HIV treatment [25], gamma gap decreases at 6 and 12 months with concomitant reductions in the staining of IgG in immunofixation studies [27,28]. Thus, while HIV widens the gamma gap, disease severity and treatment status may lead to variation in gamma gap levels, influencing sensitivity.

Similar to HIV, the gamma gap demonstrated a high specificity for HCV infection. The mechanism for this specificity could also be related to B cell activation [30,31] in addition to reduced albumin synthesis secondary to liver disease and poor nutrition [26]. Like HIV, disease severity and treatment status affect gamma gap levels and may contribute to its poor sensitivity. With B cell activation, serum IgG and gamma globulin are not only higher in patients with HCV compared to alcoholics and healthy controls [32] but they are also elevated as Histology Activity Index score, grading score, and staging score of HCV patients increases [33]. After interferon therapy or pegylated interferon plus ribavirin, serum gamma-globulin and IgG are significantly reduced [33] along with a concomitant rise in albumin by as much as 4–5 g/L [34]. Thus, severity and treatment of HCV affect the gamma gap level, reducing the sensitivity of an elevated gamma gap threshold. Furthermore, we observed better performance between hepatitis C and aspartate aminotransferase or alanine aminotransferase, which are both components of the comprehensive metabolic panel. This suggests that the gamma gap maybe less useful than these other two items.

The gamma gap had both low specificity and sensitivity for MGUS. As opposed to HIV and HCV, which involve polyclonal activation of humoral immunity, MGUS is derived from a dysplastic condition and monoclonal gammopathy. Furthermore, MGUS is at the most stable end of the multiple myeloma spectrum, proliferating at a low rate until a second “hit” triggers malignant transformation, such as IL-6 stimulation, which has been shown to increase the amount of the monoclonal protein produced by plasma cells and inhibit albumin synthesis in the liver simultaneously [35–38]. Moreover, across the spectrum of plasma cell dyscrasias, MGUS has the lowest M-protein concentration (M-spike), a direct contributor to the gamma gap. Thus, its minimal severity may not be sufficient to mount an elevated gamma gap. This is further supported by significantly less production of IgG, IgA, and IgM as well as minimal reduction in albumin synthesis in MGUS compared to smoldering multiple myeloma and multiple myeloma [39]. As such, gamma gap is a poor proxy for this disease and alone does not provide sufficient evidence for further MGUS screening. Notably, there is no recommended treatment for MGUS to prevent or delay transformation to multiple myeloma and related diseases, and thus the value of screening for MGUS is unclear. Thus, the gamma gap is not a cost effective screening tool.

Altogether, our study provides a framework for interpreting the gamma gap in an ambulatory, community-based population. Our study shows that the gamma gap is not a sensitive test and thus cannot be used to exclude any of these three diseases. While an elevated gamma gap of 4 g/dL or greater is associated with HIV and HCV, no level of the gamma gap can effectively rule in or rule out MGUS. Thus, for HIV, clinicians should continue to screen all persons aged 13 to 64 years who present to a healthcare provider for HIV, irrespective of the presence of risk factors per the revised 2006 CDC recommendations [40], a recommendation that is congruent with high prevalence ratio even in the lower gamma gap threshold. In the presence of an elevated gamma gap of 4 g/dL or greater, it would be even more prudent to screen for HIV. Similarly, for HCV, AASLD-ISDA recommended screening for HCV based on birth cohort, risk behaviors, risk exposures, and other co-infection such as HIV [41], which should be continued regardless of the gamma gap level.

In contrast, electrophoresis and free light chains should not be performed on the basis of gamma gap alone, regardless of its threshold and in the absence of additional clinical data. This conclusion is confirmed by a clinical decision rule developed by Thakkinstain et al [3] that combines age, gender, gamma gap, hemoglobin, and eGFR to whether or not to proceed with electrophoresis for MGUS screening.

There are several limitations in our study that warrant comment. First, each of these conditions was only measured in a subset of NHANES participants with limited overlap. As a result, we could not compare performance between diseases. Second, we did not have details related to temporality, severity, duration of disease, or treatment status. It is unknown whether these pathologic features influence levels of gamma globulinemia. Third, HIV and MGUS were only assessed in age groups at higher risk for these conditions. As a result, performance of these tests should be interpreted in the context of their NHANES populations. Fourth, the NHANES include a community-based, ambulatory population representative of the US adult population. However, sicker patients with multiple comorbidities as well as with rare conditions in the US, such as autoimmune conditions (e.g. Sjogren’s or lupus) or chronic infections (e.g. malaria or leishmaniasis) that may be associated with polygammopathies are not well-represented in our study. Additional research in hospitalized patients is warranted to determine test performance in settings with more complex and advanced disease. A higher prevalence of other inflammatory conditions in a reference group (“false positives”) would reduce the specificity of the gamma gap for any particular disease. Finally, we were unable to examine multiple myeloma as this diagnosis was not available in our dataset.

This study has a number of important strengths. First, we utilized a large, representative population of the US. Second, the NHANES includes high quality, standardized measures of three conditions most often associated with the gamma gap. Third, our ambulatory population reflects a setting where the gamma gap may be incidentally found in clinical practice. As a result, our study provides meaningful guidance on whether a positive gamma gap should be followed by further testing.

In conclusion, the gamma gap, as defined by a 4 g/dL threshold, is not a sensitive test to rule out HIV, HCV, MGUS. However, a positive gamma gap does strongly suggest HIV or HCV, but not MGUS. As a result, in clinical settings where HIV or HCV may be relevant, subsequent testing is reasonable, but testing for MGUS (e.g. via electrophoresis) is not warranted in absence of additional clinical indicators. Subsequent research should evaluate factors influencing the gamma gap, such as disease severity and treatment status. While there is no evidence that a gamma gap be performed for the sole purpose of screening, given the high availability and low cost of this test, further studies should examine the role for the gamma gap as a means of improving clinical decision making in the context of additional clinical data as well as the hospital setting.

## Supporting information

The supporting information file includes gamma gap thresholds needed to achieve different performance levels for HIV, HCV, and MGUS (**S1–3 Tables**) as well as receiver operating curves for HIV, HCV, and MGUS (**S1–3 Figs**). This file also compares the area under the curves between gamma gap and other features of the comprehensive metabolic panel (**S4 Table**).

## Supporting information

S1 Fig (DOCX)

S2 Fig (DOCX)

S3 Fig (DOCX)

S1 Table (DOCX)

S2 Table (DOCX)

S3 Table (DOCX)

S4 Table (DOCX)

## References

[1] Feldman LS, Shihab HM, Thiemann D, Yeh H-C, Ardolino M, Mandell S, et al Impact of providing fee data on laboratory test ordering: a controlled clinical trial. JAMA Intern Med. 2013;173: 903–908. 10.1001/jamainternmed.2013.232 23588900

[2] Vavricka SR, Burri E, Beglinger C, Degen L, Manz M. Serum protein electrophoresis: an underused but very useful test. Digestion. 2009;79: 203–210. 10.1159/000212077 19365122

[3] Thakkinstian A, Tran H, Reeves G, Murch S, Attia J. A clinical decision rule to aid ordering of serum and urine protein electrophoresis for case-finding of paraproteins in hospitalized inpatients. J Gen Intern Med. 2008;23: 1688–1692. 10.1007/s11606-008-0712-z 18665429 PMC2533374

[4] Juraschek SP, Moliterno AR, Checkley W, Miller ER. The Gamma Gap and All-Cause Mortality. PLoS ONE. 2015;10: e0143494 10.1371/journal.pone.0143494 26629820 PMC4668045

[5] Salt HB. Serum globulin fractions in chronic rheumatic diseases; an electrophoretic study. Clin Chem. 1956;2: 35–44. 13284964

[6] Suh B, Park S, Shin DW, Yun JM, Keam B, Yang H-K, et al Low albumin-to-globulin ratio associated with cancer incidence and mortality in generally healthy adults. Ann Oncol. 2014;25: 2260–2266. 10.1093/annonc/mdu274 25057172

[7] Dispenzieri A, Gertz MA, Therneau TM, Kyle RA. Retrospective cohort study of 148 patients with polyclonal gammopathy. Mayo Clin Proc. 2001;76: 476–487. 10.4065/76.5.476 11357794

[8] O’Connell TX, Horita TJ, Kasravi B. Understanding and interpreting serum protein electrophoresis. Am Fam Physician. 2005;71: 105–112. 15663032

[9] Yang M, Xie L, Liu X, Hao Q, Jiang J, Dong B. The gamma gap predicts 4-year all-cause mortality among nonagenarians and centenarians. Sci Rep. 2018;8: 1046 10.1038/s41598-018-19534-4 29348636 PMC5773485

[10] Loprinzi PD, Addoh O. The gamma gap and all-cause mortality risk: considerations of physical activity. Int J Clin Pract. 2016;70: 625–629. 10.1111/ijcp.12817 27292974

[11] Pirrucello J. Gamma Gap [Internet]. [cited 8 Mar 2019]. Available: http://james.pirruccello.us/index.php?title=Gamma_gap

[12] Clarke K, Dobro S, Brandes L. Always Work Up a Significant Globulin Gap [Abstract]. Hospital Medicine. Available: https://www.shmabstracts.com/abstract/always-work-up-a-significant-globulin-gap/

[13] Stohl W, Kenol B, Kelly A, Ananth Correa A, Panush R. Elevated Serum Globulin Gap As a Reliable and Cost-Savings Marker of Inflammation in Patients with Systemic Rheumatic Diseases [Abstract]. Arthritis Rheum. 2018;70 Available: https://acrabstracts.org/abstract/elevated-serum-globulin-gap-as-a-reliable-and-cost-savings-marker-of-inflammation-in-patients-with-systemic-rheumatic-diseases/10.1016/j.semarthrit.2019.05.001 31153707

[14] Wheeler D. VA Report: The Elevated Protein Gap. 2016; Available: https://ucsfmed.wordpress.com/2016/02/29/va-report-the-elevated-protein-gap/

[15] Dupuis M, Zhiguo Li Z, Tuchman S, Kang Y. The Gamma Gap: A Point-of-Care Test That Correlates with Disease Burden and Treatment Response in Multiple Myeloma. Blood. 2017;130: 4407. 10.1200/JOP.19.00517 PMC7427420 32240071

[16] Hughes M, Davidson DF, McColl M. Outcomes of discretionary laboratory requesting of serum protein electrophoresis. Ann Clin Biochem. 2006;43: 372–374. 10.1258/000456306778520133 17022879

[17] Malacrida V, De Francesco D, Banfi G, Porta FA, Riches PG. Laboratory investigation of monoclonal gammopathy during 10 years of screening in a general hospital. J Clin Pathol. 1987;40: 793–797. 10.1136/jcp.40.7.793 3114329 PMC1141100

[18] NHANES 1999–2014: Standard Biochemistry Profile & Hormones Data Documentation, Codebook, and Frequencies [Internet]. Available: wwwn.cdc.gov/Nchs/Nhanes

[19] NHANES 1999–2014: HIV Antibody Test, CD4+ T Lymphocytes & CD8+ T Cells Data Documentation, Codebook, and Frequencies [Internet]. Available: wwwn.cdc.gov/Nchs/Nhanes

[20] NHANES 1999–2004: Hepatitis B: Core Antibody & Surface Antigen; Hepatitis C: Confirmed Antibody & RNA (HCV-RNA); Hepatitis D Antibody Data Documentation, Codebook, and Frequencies [Internet]. Available: wwwn.cdc.gov/Nchs/Nhanes/

[21] NHANES 2005–2014: Hepatitis C: Confirmed Antibody, RNA (HCV-RNA), & Genotype Data Documentation, Codebook, and Frequencies [Internet]. Available: https://wwwn.cdc.gov/Nchs/Nhanes

[22] NHANES 1999–2004: Monoclonal gammopathy of undetermined significance (MGUS) (Surplus) Data Documentation, Codebook, and Frequencies [Internet]. Available: wwwn.cdc.gov/Nchs/Nhanes

[23] Shirai A, Cosentino M, Leitman-Klinman SF, Klinman DM. Human immunodeficiency virus infection induces both polyclonal and virus-specific B cell activation. J Clin Invest. 1992;89: 561–566. 10.1172/JCI115621 1737846 PMC442888

[24] De Milito A, Nilsson A, Titanji K, Thorstensson R, Reizenstein E, Narita M, et al Mechanisms of hypergammaglobulinemia and impaired antigen-specific humoral immunity in HIV-1 infection. Blood. 2004;103: 2180–2186. 10.1182/blood-2003-07-2375 14604962

[25] Serpa J, Haque D, Valayam J, Breaux K, Rodriguez-Barradas MC. Effect of combination antiretroviral treatment on total protein and calculated globulin levels among HIV-infected patients. Int J Infect Dis. 2010;14 Suppl 3: e41–44. 10.1016/j.ijid.2009.10.007 20137993

[26] Scherzer R, Heymsfield SB, Rimland D, Powderly WG, Tien PC, Bacchetti P, et al Association of serum albumin and aspartate transaminase with 5-year all-cause mortality in HIV/hepatitis C virus coinfection and HIV monoinfection. AIDS. 2017;31: 71–79. 10.1097/QAD.0000000000001278 27677166 PMC5127775

[27] Adedeji AL, Adenikinju RO, Ajele JO, Olawoye TL. Serum protein electrophoresis under effective control of HIV-1 disease progression. EXCLI J. 2014;13: 761–771. 26417299 PMC4464463

[28] Konstantinopoulos PA, Dezube BJ, Pantanowitz L, Horowitz GL, Beckwith BA. Protein electrophoresis and immunoglobulin analysis in HIV-infected patients. Am J Clin Pathol. 2007;128: 596–603. 10.1309/QWTQFGA9FXN02YME 17875511

[29] Redgrave BE, Stone SF, French M a. H, Krueger R, James IR, Price P. The effect of combination antiretroviral therapy on CD5 B-cells, B-cell activation and hypergammaglobulinaemia in HIV-1-infected patients. HIV Med. 2005;6: 307–312. 10.1111/j.1468-1293.2005.00312.x 16156877

[30] Sugalski JM, Rodriguez B, Moir S, Anthony DD. Peripheral blood B cell subset skewing is associated with altered cell cycling and intrinsic resistance to apoptosis and reflects a state of immune activation in chronic hepatitis C virus infection. J Immunol. 2010;185: 3019–3027. 10.4049/jimmunol.1000879 20656924 PMC3805966

[31] Cornella SL, Stine JG, Kelly V, Caldwell SH, Shah NL. Persistence of mixed cryoglobulinemia despite cure of hepatitis C with new oral antiviral therapy including direct-acting antiviral sofosbuvir: A case series. Postgrad Med. 2015;127: 413–417. 10.1080/00325481.2015.1021660 25746436

[32] Gonzàlez-Quintela A, Alende MR, Gamallo R, Gonzàlez-Gil P, López-Ben S, Tomé S, et al Serum immunoglobulins (IgG, IgA, IgM) in chronic hepatitis C. A comparison with non-cirrhotic alcoholic liver disease. Hepatogastroenterology. 2003;50: 2121–2126. 14696478

[33] Maruyama S, Hirayama C, Horie Y, Yorozu K, Maeda K, Inoue M, et al Serum immunoglobulins in patients with chronic hepatitis C: a surrogate marker of disease severity and treatment outcome. Hepatogastroenterology. 2007;54: 493–498. 17523306

[34] Zhang G-L, Chen Y-M, Zhang T, Cai Q-X, Zhang X-H, Zhao Z-X, et al Favorable Outcomes of Chinese HCV-Related Cirrhotic Patients with Sustained Virological Response after Pegylated Interferon Plus Ribavirin Treatment. Biomed Res Int. 2017;2017: 8061091 10.1155/2017/8061091 28232944 PMC5292367

[35] Kádár K, Wolf K, Tábori J, Karádi I, Várkonyi J. The albumin and monoclonal protein ratio as prognostic marker for multiple myeloma in the era of novel agents. Pathol Oncol Res. 2012;18: 557–561. 10.1007/s12253-012-9506-z 22314327

[36] Kim JE, Yoo C, Lee DH, Kim S-W, Lee J-S, Suh C. Serum albumin level is a significant prognostic factor reflecting disease severity in symptomatic multiple myeloma. Ann Hematol. 2010;89: 391–397. 10.1007/s00277-009-0841-4 19844712

[37] Waxman AJ, Mick R, Garfall AL, Cohen A, Vogl DT, Stadtmauer EA, et al Classifying ultra-high risk smoldering myeloma. Leukemia. 2015;29: 751–753. 10.1038/leu.2014.313 25371175

[38] Bladé J, Rosiñol L, Cibeira MT, de Larrea CF. Pathogenesis and progression of monoclonal gammopathy of undetermined significance. Leukemia. 2008;22: 1651–1657. 10.1038/leu.2008.203 18668131

[39] Cesana C, Klersy C, Barbarano L, Nosari AM, Crugnola M, Pungolino E, et al Prognostic factors for malignant transformation in monoclonal gammopathy of undetermined significance and smoldering multiple myeloma. J Clin Oncol. 2002;20: 1625–1634. 10.1200/JCO.2002.20.6.1625 11896113

[40] Branson BM, Handsfield HH, Lampe MA, Janssen RS, Taylor AW, Lyss SB, et al Revised recommendations for HIV testing of adults, adolescents, and pregnant women in health-care settings. MMWR Recomm Rep. 2006;55: 1–17; quiz CE1-4. 16988643

[41] AASLD/IDSA HCV Guidance Panel. Hepatitis C guidance: AASLD-IDSA recommendations for testing, managing, and treating adults infected with hepatitis C virus. Hepatology. 2015;62: 932–954. 10.1002/hep.27950 26111063

