# Supplementary figures and images for: Gamma gap thresholds and HIV, hepatitis C, and monoclonal gammopathy
Source: PLoS One. 2020 Jan 15;15(1):e0224977. doi: 10.1371/journal.pone.0224977 (PMC6961927; doi:10.1371/journal.pone.0224977)

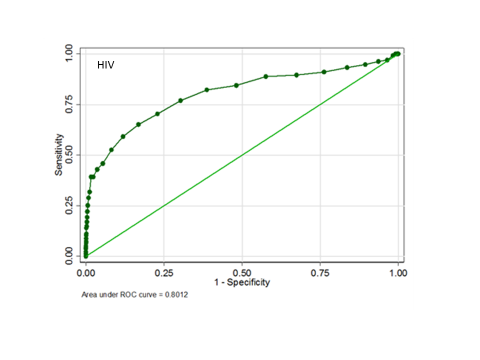


**S1 Figure.** Receiver-Operating Characteristic (ROC) curve for HIV.

Supplement: S1 Fig — (DOCX) [file pone.0224977.s001.docx]

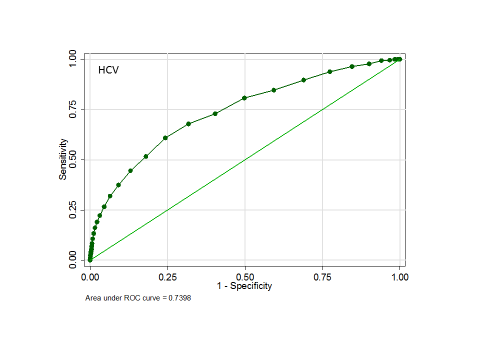


**S2 Figure.** Receiver-Operating Characteristic (ROC) curve for HCV.

Supplement: S2 Fig — (DOCX) [file pone.0224977.s002.docx]

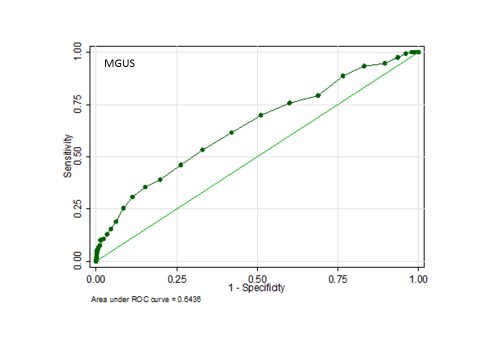


**S3 Figure.** Receiver-Operating Characteristic (ROC) curve for MGUS.

Supplement: S3 Fig — (DOCX) [file pone.0224977.s003.docx]

**S1 Table. Gamma gap thresholds needed to achieve difference performance levels for HIV, N = 25,680**


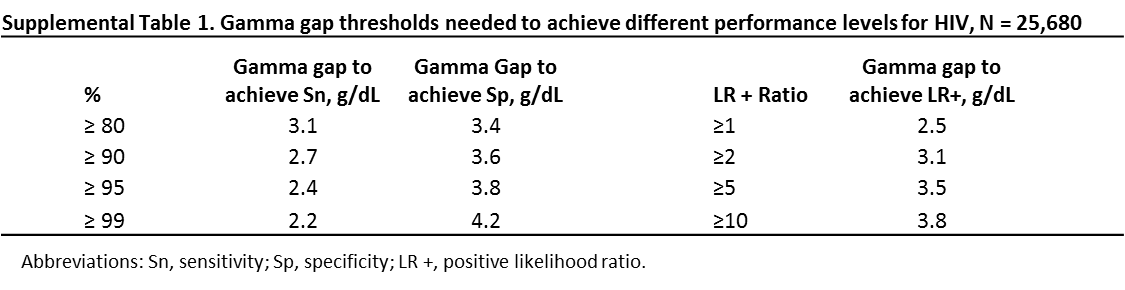

Supplement: S1 Table — (DOCX) [file pone.0224977.s004.docx]

**S2 Table. Gamma gap thresholds needed to achieve difference performance levels for HCV, N = 45,134**


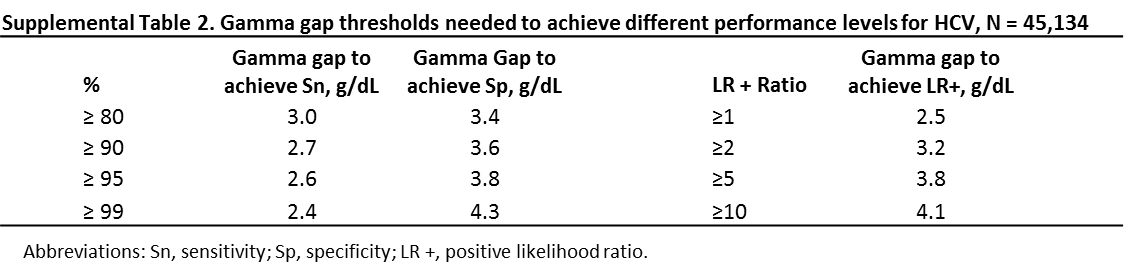

Supplement: S2 Table — (DOCX) [file pone.0224977.s005.docx]

**S3 Table. Gamma gap thresholds needed to achieve difference performance levels for MGUS, N = 6,118**


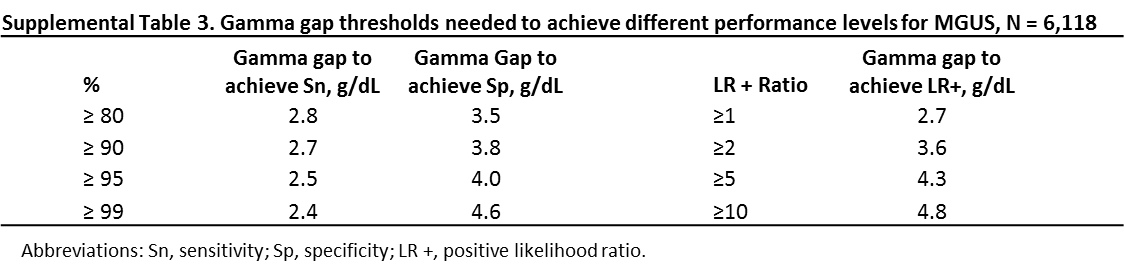

Supplement: S3 Table — (DOCX) [file pone.0224977.s006.docx]
