# Supplementary material for: Gamma gap thresholds and HIV, hepatitis C, and monoclonal gammopathy
Source: PLoS One. 2020 Jan 15;15(1):e0224977. doi: 10.1371/journal.pone.0224977 (PMC6961927; doi:10.1371/journal.pone.0224977)
Supplement: S4 Table — (DOCX) [file pone.0224977.s007.docx]

| **S4 Table. Comparison of area under the curves for gamma gap with other features of the comprehensive metabolic panel, area under the curve (95% CI)** | | | |
| --- | --- | --- | --- |
|  | **HIV** | **Hepatitis C** | **MGUS** |
| Gamma gap, g/dL | 0.80 (0.75, 0.85) | 0.74 (0.72, 0.76) | 0.64 (0.60, 0.69) |
| Total protein, g/dL | 0.77 (0.72, 0.81) | 0.63 (0.60, 0.66) | 0.58 (0.54, 0.63) |
| Albumin, g/dL | 0.38 (0.33, 0.43) | 0.33 (0.31, 0.36) | 0.38 (0.34, 0.43) |
| Total calcium, mg/dL | 0.46 (0.41, 0.51) | 0.42 (0.40, 0.45) | 0.48 (0.44, 0.52) |
| Aspartate aminotransferase, U/L | 0.70 (0.65, 0.74) | 0.89 (0.87, 0.90) | 0.48 (0.44, 0.52) |
| Alanine aminotransferase, U/L | 0.61 (0.56, 0.66) | 0.88 (0.86, 0.89) | 0.44 (0.40, 0.48) |
| Alkaline phosphatase, U/L | 0.58 (0.53, 0.63) | 0.49 (0.47, 0.52) | 0.47 (0.43, 0.52) |
| Total bilirubin, mg/dL | 0.46 (0.41, 0.51) | 0.53 (0.51, 0.56) | 0.51 (0.47, 0.55) |
